# In-Game Play Behaviours during an Applied Video Game for Anxiety Prevention Predict Successful Intervention Outcomes

**DOI:** 10.1007/s10862-018-9684-4

**Published:** 2018-06-11

**Authors:** Aniek Wols, Anna Lichtwarck-Aschoff, Elke A. Schoneveld, Isabela Granic

**Affiliations:** Behavioural Science Institute, Radboud University, P.O. BOX 9104, 6500 HE Nijmegen, The Netherlands

**Keywords:** Anxiety, Prevention, Children, Applied games, Mechanisms

## Abstract

Anxiety disorder is the most prevalent and frequently diagnosed disorder in youth, and associated with serious negative health outcomes. Our most effective prevention programs, however, have several limitations. These limitations can be addressed using game-based interventions. Results from two randomized controlled trials on the video game *MindLight* show improvements in anxiety that are maintained up to 6 months. The game was designed based on evidence-based therapeutic techniques; however, it is unclear if children’s engagement with these techniques actually predict improvements in anxiety symptoms. An important advantage of game-based interventions is that they provide excellent opportunities to isolate therapeutic action mechanisms and test their impact on intervention outcomes. In the current study, on-screen videotaped output while playing *MindLight* was coded and analysed for forty-three 8 to 12-year old children with elevated levels of anxiety. Results showed that changes in in-game play behaviours representing therapeutic exposure techniques predicted improvements in anxiety symptoms 3 months later (when children had not played the game for 3 months). The current study is a first step towards identifying and validating game mechanics that can be used in new applied games to target anxiety symptoms or other psychopathologies with the same underlying deficits.

Anxiety disorders are the earliest form of psychopathology to emerge in childhood, the most prevalent and frequently diagnosed disorders in youth (Beesdo et al. 2009), and associated with adverse health outcomes (Brent et al. 1986; Essau 2003; Kessler et al. 1996; Pine et al. 1998; Weissman et al. 1999; Woodward and Fergusson 2001). Beyond the numbers for clinical diagnoses, sub-clinical levels of anxiety symptoms are estimated at 40% in children (Muris et al. 2000a), which increase the risk for full-blown anxiety disorders at older ages. Currently, programs based on Cognitive Behavioural Therapy (CBT) have been shown to be most effective (Butler et al. 2006; Fisak Jr et al. 2011; In-Albon and Schneider 2007; Kendall 2011; Mychailszyn et al. 2012). However, CBT outcomes are mixed and effect sizes are small to moderate (Fisak Jr et al. 2011; Mychailszyn et al. 2012).

These disappointing outcomes of CBT-based indicated prevention programs might be related to limitations regarding the way in which interventions are delivered, rather than the therapeutic principles on which they are based (Granic et al. 2014; Kazdin 2011). These limitations include a didactic-based approach that might not be engaging and motivating for some children (Gosch et al. 2006), few opportunities to practice newly-acquired knowledge, non-adherence of practitioners to the protocol (Eichstedt et al. 2014), and low accessibility and high costs of interventions (Collins et al. 2004; Farmer et al. 1999; Kataoka et al. 2002).

The aforementioned limitations can be addressed by using game-based interventions (see Granic et al. 2014 for full discussion; Rideout et al. 2010; Olson 2010). On this basis, the video game *MindLight* (GainPlay Studio 2014) was developed in order to prevent the escalation of anxiety in at-risk children. The game incorporates evidence-based techniques by translating these techniques into game mechanics. Game mechanics are the actions in the game that are designed for the player to repeat over and over; they are the vehicles by which certain skills are trained (see Table 1). Results from an indicated randomized controlled trial (RCT; Schoneveld et al. 2016) showed that after playing 5 sessions of *MindLight*, at-risk children showed significant reductions in anxiety symptoms by 3-months follow-up. In addition, a second indicated RCT showed that playing *MindLight* for 6 sessions is as effective as a CBT-based indicated prevention program (i.e., Coping Cat; van Starrenburg et al. 2017) in reducing anxiety symptoms from pre- to post-measurement, including the 3- and 6-months follow-up (Schoneveld et al. 2018).^1^ Finally, results from a third study showed that compared to online CBT-based psychoeducation, playing *MindLight* resulted in similar improvements in anxiety symptoms and state anxiety in response to a (social and cognitive) stressor (Tsui et al. 2018, manuscript submitted for publication).

**Table 1:** Overview of the evidence-based principles that are translated into game mechanics in *MindLight* and the specific in-game play behaviours that are indicative of these mechanics

Evidence-based principle	Game mechanic	In-game play behaviours	
		Engaged	Avoidant/safety
Relaxation	Neurofeedback	Bright mindlight	No mindlight
Exposure	Approach fear events	Exploration Decloak/attack fear events Defeat Pick up coins	Turn on ceiling light Hide in chest Inactivity
Attention bias modification	Attention bias puzzles	Solve puzzle	

These results are promising, but it remains unclear whether the means by which children improve in anxiety symptoms are through the game mechanics that were explicitly designed into *MindLight.* Investigating such in-game processes is extremely valuable, not only because it gives insight into who will benefit most from the game, but also into which game mechanics are most important for anxiety symptom-reduction more generally (and those that are in need of further development). By testing the effect of the game mechanics in *MindLight*, the current study provides a first step towards building a toolbox of validated game mechanics. In turn, those validated game mechanics that are able to change causal processes associated with the development and maintenance of anxiety could be used in new applied games targeting anxiety symptoms or other psychopathologies with the same underlying deficits.

## MindLight

*MindLight* is a video game for children 8 to 12 years of age, designed and developed in a cross-disciplinary collaboration among developmental psychologists, clinicians, game designers, and children themselves. In the game, the player is in grandma’s dark and decrepit mansion and needs to save her from evil forces by chasing away or uncovering “fear events” and solving puzzles. The player plays through the game by using his/her “mindlight”, a beam of light at the end of an antenna attached to the magical hat the avatar is wearing. The player needs this light in order to move through the dark game environment.

*MindLight* incorporates three evidence-based techniques based on cognitive-behavioural principles: relaxation through neurofeedback training (Price and Budzynski 2009), exposure training (Feske and Chambless 1995), and attention bias modification (Bar-Haim 2010; Bar-Haim et al. 2011). These techniques have been repeatedly shown to address causal processes associated with the development and maintenance of anxiety such as avoidance and negative attention bias (Mathews and MacLeod 2005; Weersing et al. 2012). In *MindLight*, the three techniques are translated into specific game mechanics: neurofeedback, approaching fear events, and attention bias modification puzzles (see Table 1), together aiming at teaching children how to cope with anxiety in a playful manner. Below we explain these techniques and game mechanics in more detail. More information can also be found in Appendix 1.

The first technique incorporated in *MindLight* is relaxation training through the use of neurofeedback. In conventional neurofeedback training individuals are presented with real-time electroencephalogram (EEG) recordings from their brain and guided through relaxation exercises in order to keep their EEG waves consistent with identified proxies of relaxation (i.e., reduction in relative beta power and increase in relative alpha power; Price and Budzynski 2009). In *MindLight*, the player wears an EEG headset with one (dry) sensor touching the forehead and one reference point located in the clip attached to the left earlobe. The headset detects EEG signals and converts them into a continuous data stream representing relaxation (Johnstone et al. 2012). This data stream is fed into the game and controls the amount of light that shines in the game. The brightness of players’ mindlight is proportional to the strength of the real-time relaxation of the player. When the player becomes more relaxed the mindlight becomes brighter, providing more light in the game environment. This way, players get feedback on their level of relaxation. Thus, relaxation is trained through the neurofeedback game mechanic in *MindLight* (see Table 1).

The second technique incorporated in *MindLight* is exposure training. There are fear events (i.e., fearful obstacles) in the game and by shining one’s mindlight on them they can be chased away or uncovered. Some fear events will then turn into a friendly kitten that follows the player and that reminds the player of past fears conquered. Other fear events turn into a benign object or animal and reward the player with a coin that is needed to unlock puzzles (see below). To play through the game, the fear events need to be approached. However, a player can also escape them (temporally) by hiding inside one of the chests or by turning on a ceiling light (both instances of safety behaviours). Thus, exposure training is done through the fear events in the game (see Table 1).

The third technique incorporated in *MindLight* is attention bias modification (ABM). Attentional biases characterized by a hyper attention towards potential threats play an important role in the development and maintenance of anxiety disorders (Muris 2016; Muris and Field 2008). With ABM-training, the attentional bias towards threats is retrained such that individuals pay more attention to positive stimuli rather than to negative stimuli (Bar-Haim 2010; Bar-Haim et al. 2011). Conventional ABM-procedures have used a modified version of the dot-probe task or a visual search task and have been shown to reduce anxiety (at least in the short term; Bar-Haim 2010; Bar-Haim et al. 2011; Hakamata et al. 2010). In *MindLight*, gaming elements have been added to the main principles of the dot-probe training. In the ABM puzzles, the player learns to focus on and attend to portraits of happy faces rather than threatening faces. Upon completion of the puzzle, the lights will turn back on in that particular room. Thus, ABM-training is done through the ABM-puzzles in *MindLight* (see Table 1)*.*

## In-Game Play Behaviours

Although the game was designed to provide children with repeated training opportunities to learn emotion-regulation strategies, there is also variability in how much children actually practice these skills. This variability is largely a function of the design of the game, which allows children to play at their own pace and explore and progress through the game in a variety of ways that foster a sense of autonomy and fun. For example, some children may be too afraid to move through the game at first, and will hide in chests to avoid the fearful stimuli, while others might prefer to explore a great deal more from the outset. These play-pattern differences may lead to differences in the amount of opportunities to practice the relaxation skills or to encounter fear events further in the game.

There are several specific in-game play behaviours that are most relevant to the intervention goals of *MindLight* (see Table 1)*.* These behaviours can be classified into two types: “engaged” and “avoidant/safety” in-game play behaviours. Clinical research has shown that avoidant and/or safety behaviours are important maintenance processes for anxiety symptoms (Clark 1999; McManus et al. 2008; Salkovskis et al. 1999; Salkovskis et al. 1996), and that reducing avoidant and/or safety behaviours and increasing engagement with the treatment are predictive of better treatment outcomes (e.g., Glenn et al. 2013; Morgan and Raffle 1999; Salkovskis et al. 1999).

While playing *MindLight*, children can show in-game play behaviours that indicate engagement versus avoidant/safety behaviour. To be successful at *MindLight,* children need to show engaged in-game play behaviours, which will lead to a higher “dosage”, or more practice, with the game mechanics. Avoidant/safety in-game play behaviours limit that “dosage”. Differences in the dosage may result in anxiety symptom-reduction for some children and no anxiety symptom-reduction for other children based on *how* they played the game.

Table 1 presents an overview of the in-game play behaviours categorized as “engaged” versus “avoidant/safety” and how these behaviours relate to the game mechanics and cognitive-behavioural principles that are incorporated in *MindLight*. In the “engaged” category, all behaviours represent experiences that support players’ practice of relaxation, exposure, and modifying attention biases. For example, by exploring children are more exposed to fear events, which they can chase away or decloak and from which they can learn how to regulate their anxiety in the face of perceived threat. By solving puzzles they learn to focus more on the positive faces than on the negative ones. The “avoidant/safety” category represents behaviours that interfere with the intervention goals in *MindLight*. For example, hiding inside a chest or being inactive reduces exposure to fear events in the game. Turning on ceiling lights minimizes relaxation training, because under the ceiling light monsters are less likely to show up and the child needs to rely less on his or her own mindlight to brighten the environment.

## Design and Hypotheses

In the current study, participants were children with elevated levels of anxiety that participated in a RCT to test the effect of 6 play-sessions of *MindLight* compared to CBT (Schoneveld et al. 2018). Only children from the *MindLight* group were included in the current study. Engaged and avoidant/safety in-game play behaviours were coded for the first and last play-sessions to examine 1) how pretest anxiety scores were related to in-game play behaviours during the first play-session, and 2) whether changes in in-game play behaviours from the first to the last play-session predicted changes in anxiety symptoms at the 3-months follow-up assessment. We hypothesized that a) higher pretest anxiety scores would be related to less engaged, and more avoidant/safety, in-game play behaviours during the first play-session, and b) increases in engaged behaviours and decreases in avoidant/safety behaviours from the first to the last play-session would predict reductions in anxiety symptoms at the 3-months follow-up assessment.

## Method

### Participants

Forty-three children (20 boys, 23 girls) with elevated levels of anxiety participated in the current study. Participants’ age ranged from 8.17 to 12.65 years (*M* = 9.94, *SD* = 1.14) at pretest. All children attended primary school in the east of the Netherlands and 97.7% were born in the Netherlands

### Procedure

The current study was part of a 2-armed indicated prevention RCT (Schoneveld et al. 2018) which has been approved by the ethics committee of the Faculty of Social Sciences. Within this RCT, the effect of *MindLight* on anxiety symptoms is compared to the effect of the CBT-program *Coping Cat* (Kendall and Hedtke 2006; van Starrenburg et al. 2017). Participants for this RCT were selected based on their score on the child-version of the Spence Children’s Anxiety Scale (SCAS-C; Spence 1998). Children were included with a total SCAS-score one standard deviation above the mean *or* when they scored one standard deviation above the mean on two subscales of the SCAS (not including the obsessive compulsive disorder subscale). Children that already received mental health care were excluded. Seven hundred and ninety-one children from eight primary schools filled out the screening. Two hundred and eight children met the inclusion criteria, of which 174 children agreed to participate in the study. Eighty-six children were randomly allocated to play *MindLight.* Seventy-two children completed the *MindLight-*intervention.

Participants played *MindLight* (Version 1.0.1; 2014) for a total of approximately 6 hours. Gameplay was broken down into six 1-hour sessions spread out over a 6-week period. *MindLight* sessions took place under supervision of two research assistants at the children’s own school after school hours. *MindLight* was played on 15.6-in. ASUS X552CL-SX033H laptops, using a MindWave headset (Version 1.1.23, Neurosky Inc. 2011; Johnstone et al. 2012) and a Xbox 360 controller. Children and their parents filled out several questionnaires including the Spence Children’s Anxiety Scale (Spence 1998) 2 weeks before and after playing *MindLight*, at 3-months and 6-months follow-up (when children had not played the game for 3 respectively 6 months).

Before coding the in-game data for the current study, only children that had completed both pre- and post-measurements of anxiety symptoms were included. In addition, only children with (almost) complete video data were selected: children with less than 4 recorded and/or attended play-sessions, with no recorded first and/or last play-session, with only a short video of the first session (i.e., 6 and 9 min), or with significant disturbances^2^ during gameplay were excluded. In total, video data of 43 children were coded and analysed. Children that were excluded did not significantly differ from the children that were included with respect to their sex, age and pretest anxiety scores (χ^2^(1, *N* = 77) = 0.53, *p* = .466; *t*(75) = −0.60, *p* = .551; and *t*(75) = 0.94, *p* = .351, respectively).

### Measures

#### Anxiety Symptoms

The Spence Children’s Anxiety Scale (SCAS-C; Spence 1997, 1998) was used to assess anxiety symptoms before and after playing *MindLight*. This scale consisted of 44 items where children are asked how often (i.e., never, sometimes, often, always; scored as 0–3) they experience symptoms of six DSM-IV defined anxiety disorders, namely separation anxiety disorder (6 items), social phobia (6 items), panic disorder and agoraphobia (9 items), physical injury fears (5 items), generalized anxiety disorder (6 items), and obsessive-compulsive disorder (6 items). Six items were positive filler items to reduce negative response bias. Mean anxiety scores were calculated over the 38 anxiety items and used in the analyses. The SCAS-C is a reliable and valid measure (Birmaher et al. 1997; Muris et al. 1998; Muris et al. 2000b; Reynolds and Richmond 1978) and in the current study internal consistency was good; Cronbach’s alpha = .89 for pretest and .90 for posttest.

#### Coding of in-Game Play Behaviours

During the *MindLight* sessions, the on-screen output was videotaped using Fraps (Version 3.5.99; 2015). This program records the exact output of the screen (i.e., what the children see on their screens while playing the game). On-screen video data was coded using observational codes with the Noldus Observer XT (Version 11.5; Noldus Information Technology 2013). Real-time in-game play behaviours during the first and last play-session^3^ were coded following the adapted version of the *MindLight Coding System* (i.e., *MCS-II*; based on Sherman 2015; see Appendix Table 6 for written guidelines of all codes) which has been developed to measure the frequency and duration of different in-game play behaviours. The *MCS-II* includes mutually exclusive codes for the: [1] type of engagement, [2] location in the game-environment, [3] presence of different kinds of fear events, and [4] brightness of the mindlight^4^ (which could be “none”, “some”, or “total”). In the current study, codes of interest included four engaged in-game play behaviours and two avoidant/safety in-game play behaviours (see Table 1). Engaged behaviours were the *frequency* of defeat and the *duration* of exploration, attempts to decloak or attack fear events (“fear attempts”), and the brightness of the mindlight. Avoidant/safety behaviours were the *frequency* of ceiling light attempts, and the *duration* of hiding inside a chest. A detailed description of all codes can be found in Appendix Table 6.

The first author trained research assistants in the use of Noldus Observer XT and the *MindLight Coding System-II*. Training took approximately 8 hours and training materials included video data from excluded cases. Coders were blind to the hypotheses. Weekly to bi-weekly recalibration training and reliability checks were conducted to monitor coding and minimize coder drift. Fifteen percent of the total amount of coded video data was independently coded by 2 or more randomly-selected coders. The average reliability was .89 kappa (range .49–1.00).

#### Strategy of Analyses

The frequency and duration of in-game play behaviours were transformed to frequencies per minute and proportions, respectively, to control for differences in duration of play-sessions. For the brightness of the mindlight, only the durations of the two extremes (“none” and “total”) were used in the analyses. Then, descriptive statistics of the study variables and Pearson correlations between 1) pretest anxiety scores and in-game play behaviours during the first play-session, and 2) in-game play behaviours during the first play-session and the last play-session were examined. Second, two hierarchical regression analyses (one for engaged and one for avoidant/safety in-game play behaviours) were performed to examine whether changes in in-game play behaviours from the first to the last play-session (i.e., differences in frequencies/proportions calculated as last minus first play-session) were related to changes in anxiety symptoms 3 months later. The pretest anxiety score was entered in the first step. The difference in frequency or proportion of the specific in-game play behaviours were entered as a set in the second step. The anxiety score at the 3-months follow-up assessment was entered as dependent variable. Before running the regression models, statistical assumptions necessary for multiple regression (i.e., normal distribution of measurement error, homoscedasticity of the variances, linearity of the model, and multicollinearity) were tested and met. Nevertheless, bootstrapping (*n* = 5000) was used to ensure accurate and valid results (and taking into account the small sample size). One participant that was an outlier (*z*-score > |3|) on both pretest and posttest anxiety was excluded from the analyses.

## Results

### Descriptive Statistics

Means and standard deviations of the study variables are presented in Table 2. Contrary to what was expected, anxiety at pretest was not significantly associated with the in-game play behaviours during the first play-session (see Table 3). In addition, age and sex were not significantly associated with anxiety at pretest nor with the different in-game play behaviours during the first play-session.

**Table 2:** Means and standard deviations (SDs) of the study variables

	Demographics	First play-session	Last play-session
Anxiety at pretest	0.91 (0.31)		
Anxiety at 3-months follow-up^a^	0.64 (0.37)		
Age	9.94 (1.15)		
Sex	.48 (.51)		
Engaged in-game play behaviours			
Mindlight - total		.53 (.10)	.46 (.13)
Exploration		.72 (.09)	.81 (.11)
Fear attempt		.38 (.16)	.25 (.20)
Defeat		0.05 (0.03)	0.07 (0.04)
Avoidant/safety in-game play behaviours			
Mindlight - none		.02 (.04)	.05 (.06)
Ceiling light attempt		0.20 (0.13)	0.07 (0.08)
Inside chest		.04 (.03)	.02 (.06)

Note. *n* = 42, ^a^ *n* = 38. All in-game play behaviours are proportions, except for ‘defeat’ and ‘ceiling light attempt’ which are frequencies

**Table 3:** Pearson correlations of anxiety at pretest, age, sex, and in-game play behaviours during the first play-session

	1	2	3	4	5	6	7	8	9
Demographics									
1. Anxiety at pretest									
2. Age	.25								
3. Sex	- .22	.01							
Engaged in-game play behaviours									
4. Mindlight - total	.12	- .04	- .08						
5. Exploration	- .03	.01	.08	.32^*^					
6. Fear attempt	- .14	.01	.12	- .02	- .42^**^				
7. Defeat	.15	- .05	.02	.18	.05	.03			
Avoidant/safety in-game play behaviours									
8. Mindlight - none	.11	.03	- .10	- .29^a^	- .14	- .28^b^	- .05		
9. Ceiling light attempt	.13	.06	.21	- .32^*^	- .46^**^	.01	.27^c^	.10	
10. Inside chest	.18	- .15	.06	- .08	- .54^***^	- .03	- .14	.07	.16

Note. * *p* < .05, ** *p* < .01, *** *p* < .001, ^a^ *p* = .067, ^*b*^ *p* = .070, ^c^ *p* = .090. *n* = 42. All in-game play behaviours are proportions, except for ‘defeat’ and ‘ceiling light attempt’ which are frequencies. Sex was coded as 0 for girls and 1 for boys. Partial correlations testing age and sex as potential suppressor variables showed similar results

Moderate to large correlations were found between several in-game play behaviours during the first play-session (see Table 3). Exploration was positively associated with total mindlight, and negatively associated with ceiling light attempts, hiding inside a chest, and fear attempts. Further, total mindlight was negatively associated with ceiling light attempts. Thus, a child that spent large amounts of time exploring the game environment showed longer periods of bright mindlight, fewer ceiling light attempts, and shorter periods of time hiding inside a chest and decloaking or attacking fear events. Longer periods of bright mindlight were associated with fewer ceiling light attempts.

Regarding the correlations between in-game play behaviours during the first and the last play-session, Table 4 shows that none of the in-game play behaviours were associated with one another over time except for the negative association between exploration during the first play-session and fear attempts during the last play-session. It might be that children that explored more during the first session defeated more fear events during the game, resulting in less fear attempts needed during the last session.

**Table 4:** Pearson correlations of in-game play behaviours during the first and last play-session

Last play-session	1	2	3	4	5	6	7
First play-session							
Engaged in-game play behaviours							
1. Mindlight - total	.05	.09	.01	.04	- .09	.04	.12
2. Exploration	- .22	.25	- .36^*^	- .07	- .09	.13	- .13
3. Fear attempt	.10	- .20	.17	.03	.06	.02	.12
4. Defeat	- .15	.15	.06	.12	.03	.03	- .19
Avoidant/safety in-game play behaviours							
5. Mindlight - none	.09	.11	- .06	.16	- .17	.12	- .13
6. Ceiling light attempt	- .05	- .05	- .14	.03	.18	- .07	.01
7. Inside chest	.20	- .07	.30^a^	.01	- .11	- .24	.18

Note. * *p* < .05, ** *p* < .01, *** *p* < .001, ^a^ *p* = .051, *n* = 42. All in-game play behaviours are proportions, except for ‘defeat’ and ‘ceiling light attempt’ which are frequencies. Partial correlations testing age and sex as potential suppressor variables showed similar results

### In-Game Play Behaviours and Improvements in Anxiety 3 Months Later

Table 5 presents hierarchical regression analyses predicting anxiety at the 3-months follow-up from the difference in in-game play behaviours from the first to the last play-session controlled for anxiety at pretest. Model statistics represent the difference in the unique proportion of variance explained by the engaged and avoidant/safety in-game play behaviours respectively, beyond the proportion of variance explained by anxiety at pretest. Anxiety at pretest explained 44.4% of the variance in anxiety at the 3-months follow-up.

**Table 5:** Hierarchical regression analyses predicting anxiety at the 3-months follow-up from the differences in in-game play behaviours from the first to the last play-session controlled for anxiety at pretest

Model	Unstandardized estimate	Standardized estimate	Bootstrap 95% CI for B		Model Statistics		
	B (*SE*)	*β*	Lower	Upper	Δ *F*	Error *df*	Δ *R*^*2*^
Anxiety pretest	0.82 (0.15)	.67^***^	0.54	1.02	28.76	36	.44^***^
Engaged in-game play behaviours					2.99	32	.15^*^
Anxiety pretest	0.90 (0.15)	.74^***^	0.60	1.17			
Difference in mindlight - total	- 0.29 (0.25)	- .13	- 0.82	0.35			
Difference in exploration	- 1.02 (0.38)	- .33^**^	- 1.80	- 0.37			
Difference in fear attempt	- 0.27 (0.19)	- .16	- 0.69	0.07			
Difference in defeat	1.22 (0.84)	.18	- 0.52	2.99			
Avoidant/safety in-game play behaviours					5.82	33	.19^**^
Anxiety pretest	0.90 (0.13)	.74^***^	0.65	1.11			
Difference in mindlight - none	0.66 (0.48)	.15	0.02	2.89			
Difference in ceiling light attempt	0.68 (0.25)	.29^*^	0.21	1.23			
Difference in inside chest	1.51 (0.58)	.28^*^	0.23	3.02			

Note. * *p* < .05, ** *p* < .01, *** *p* < .001, 5.000 bootstrap samples. Thirty-eight of the forty-two children completed the 3-months follow-up assessment. All in-game play behaviours are proportions, except for ‘defeat’ and ‘ceiling light attempt’ which are frequencies

**Table 6:** MindLight Coding System-II

Engagement behaviour codes		
These codes describe the character’s behaviour in the game.		
Name	Code	Description
Teru Story	ts	Activate this code when Teru explains things to the player character while the picture is letterboxed. The code starts when the picture becomes letterboxed and ends when the picture returns to full screen again. This code is also activated when Arty discovers the magical hat. After Teru’s story, activate the *Exploration* code before you use another code.
Defeat	d	Coded as point event. Defeat occurs when the health meter in the lower-left corner of the screen is reduced to zero heart icons. Granny will begin to hunt the character. If the character is caught by Granny, he will be incapacitated and sent back to the map room. The picture will fade out to black, and then fade in as the character revives in the map room. Activate the *Defeat* code as soon as the picture fades to black. In between, most of the times the *Exploration* code will be activated.
Inactive	in	The player character is classified as inactive if three conditions are met: (1) the character remains still and (2) there are no changes in camera angle (3) for a period of 6 s or more. Activate the code after these 6 s. NB. When the character is inside a chest, use the *Inside Chest* code. When the character is in front of a fear event, use the *Fear Attempt* code.
Exploration	ex	The player character is moving through the game-environment *or* the camera angle is being adjusted.
Ceiling light attempt	cla	The player character begins to project his mindbeam onto a ceiling light.
Ceiling light success	cls	Coded as point event. The ceiling light turns on and illuminates the surrounding environment.
Chest attempt	cha	The player character begins to project his mindbeam onto a chest. If the player is successful, a cloud of smoke will appear and the character will be transported into the chest. Consequently, when the cloud of smoke appears, immediately activate the *Inside Chest* code. When the character leaves the chest, activate the *Exploration* code. If the player is unsuccessful, the mindbeam will not be projected onto the chest anymore. Activate the appropriate engagement code (this will be the *Exploration* code).
Inside chest	ich	When the cloud of smoke appears, immediately activate the *Inside Chest* code. When the character leaves the chest, activate the *Exploration* code.
Pick up coin	coi	Coded as point event. The player character picks up a coin. NB. Sometimes you don’t see this (due to e.g., camera angle), but you will hear when a coin is picked up.
Decloak cat	cat	Coded as point event. Sometimes when the player character decloaks a (green-eyed) fear event, a cat will appear. Code when the cat appears.
Puzzle activation	pac	Coded as point event. Use this code when the player character activates the blue cog-shaped platform of a puzzle.
Puzzle attempt	pat	Start this code as soon as the mindbeam makes contact with the puzzle face. Stop the code and use the *Exploration* code when the mindbeam is no longer connected to the puzzle face.
Puzzle success	psu	Coded as point event. When the attention bias modification task within a puzzle room is completed, the room will light up. Pleasant music is audible in these illuminated rooms. Activate the *Puzzle Success* code as soon as the room lights up. NB. Also use this code when the player solves the map room puzzle multiple times.
Fear attempt	fat	The player character moves towards the fear event and/or stays in front of the fear event in order to let it disappear (this means that the player character doesn’t necessarily need to be facing the fear event). Start the code at the right distance (i.e., when you see purple spots). NB. Sometimes the player might stay in front of a monster that cannot be beaten. Do activate this code at the same distance as you would do for the other monsters.
Fear success	fsu	Coded as point event. The player character successfully decloaks the fear event.
Technical problem	2	Use this code when the picture freezes, the game is paused, when the game is restarted or when there is no connection with the mindwave (green circle is half or less). Start the code from the beginning that this technical problem appeared.
Location codes		
These codes describe the character’s location		
Name	Code	Description
Entry hall	ent	When you just entered the house, you will be in this hallway. There are two doors at both ends of the hallway (the front door which is locked and the door to the bedroom).
Bedroom	bed	Arty’s bedroom. There are, for instance, his bed, pictures on the wall, teddy bears and a box behind his bed.
Couch room	cr	This is the room you enter after the bedroom. In this room there are two red couches and on the other side of the room there are three chairs. In this room the player character learns how to turn on a ceiling light.
Hall between couch room and fear room	hb	After the couch room you walk through this hallway to the fear room. There are no doors and only one ceiling light in this hallway.
Fear practice room	fp	In this room there is a blue round carpet and the player character learns to decloak a fear event. There is one door that is locked and the other door is already open.
Box hall	bh	You enter this hallway by walking through the open door in the fear room. This hallway has a lot of boxes and one chest. The player character learns here to open a chest. This hallway leads to the map room or to the secret hallway (if you walk around the door).
Secret hall	sh	You can only enter this hallway by walking around the door between the fear room and the box hall. After a right turn the hallway will have five doors.
Map room	mp	The map room contains a map of the game environment, which is located to the left of a door flanked by two puzzle faces. This room serves two general purposes: *recuperation* and *orientation*. The player is transported back to the map room upon defeat. Alternatively, the player may return to the map room voluntarily to orient themselves within the game space.
Box room	box	In this room there are a lot of piled boxes and chairs. The puzzle in this room consists of three faces.
TV room	tv	In this rooms there are a lot of seating areas and a TV that is very noisy. The puzzle in this room consists of four faces.
Ball room	bal	In this room there are different long dining tables, flags and knights. The puzzle in this room consists of five faces.
Tree room	tre	In this room there are different gardens with a lot of trees. The puzzle in this room consists of six faces.
Fear room	fr	In this room there are several fear events and seven coins that need to be collected to activate the puzzle in the Final Room. Through this room it is possible to enter the Final Room (although it is also possible to enter the Final Room right away).
Final room	fin	After turning on the lights in all the rooms, the Final Room is unlocked. This is the hardest room in the game. If the player is successful in finding all the coins and solving the puzzle in this room, all the lights in the mansion will turn on. The player character then receives a letter from his deceased grandpa with the instruction to bring this to Granny’s room in order to save her.
Granny’s room	gr	When the player character enters this room with the letter from grandpa, the picture will be letterboxed and shows the end of the story.
Small hall	sma	Hallways are areas of the game that connect puzzle rooms. If there are no coin icons in the upper-right corner of the screen, then the character is in a hallway. There are no monsters in the small hallway.
Wide hall	wid	Hallways are areas of the game that connect puzzle rooms. If there are no coin icons in the upper-right corner of the screen, then the character is in a hallway. There are monsters in the wide hallway.
Technical problem	1	Use this code when the picture freezes, the game is paused, when the game is restarted or when there is no connection with the mindwave (green circle is half or less). Start the code from the beginning that this technical problem appeared.
Fear event presence codes		
These codes describe the presence/absence of a fear event		
Name	Code	Description
Fear on screen – No	nf	Use this code when there are no fear events or monsters visible on the screen.
Fear on screen - Yes	yf	Use this code when there are fear events or monsters visible on the screen. Indicate under modifiers the type of fear: 1). Fear event (rumbling/shadowed objects) 2). Monster with yellow eyes 3). Monster with green eyes. NB. Sometimes you know the monsters are within the screen, but you cannot see them because of, for instance, the camera angle. Do activate this code in that case.
Technical problem	4	Use this code when the picture freezes, the game is paused, when the game is restarted or when there is no connection with the mindwave (green circle is half or less). Start the code from the beginning that this technical problem appeared.
MindLight codes		
These codes describe the brightness of the mindlight. Given the variance in ambient lighting throughout the game space, both luminosity and body language should be taken into consideration to discern the mindlight codes. When, for instance, the brightness is difficult to see (due to a lot of light in the room or the camera angle), pay attention to the body posture and facial expression of the player character.		
Name	Code	Description
Pre-Teru	P	Use this code when the player character hasn’t picked up Teru yet. Start this code when the picture fades black between the overall story of the game and that you see Arty in the first hallway.
None	N	The environment around the player is almost to completely dark and/or the character’s body language is suggestive of high state anxiety; he is frowning, slouching, and crossing his arms.
Some	S	The mindlight is extended to provide a small-to-moderate range of illumination. Furthermore, the character’s posture is suggestive of a slightly anxious state; he is holding his arms close to his body and is beginning to slouch forward.
Total	T	The mindlight is extended to provide a large range of illumination. Furthermore, the character’s posture exudes confidence; he is standing erect, smiling, and his arms are held out and to the side, causing his body to take on a triangular silhouette. Another important identifying feature of total mindlight activation is the sparkle particle effect (bright flashes of light) that is visible on the ground in front of the character.
Teru occupied	O	During engagement in activities such as turning on ceiling lights, unlocking chests, and solving puzzles, the diffuse mindlight typically seen during exploration will be focused into a beam or not visible at all (for example, when walking around while making a puzzle, when inside a chest). Activate the *Teru Occupied* code following the onset of the foregoing activities.
No connection	C	Use this code when there is no connection with the mindwave (green circle is half or less).
Technical problem	3	Use this code when the picture freezes, the game is paused or when the game is restarted. Start the code from the beginning that this technical problem appeared.

We hypothesized that increases in engaged in-game play behaviours and decreases in safety/avoidant in-game play behaviours from the first to the last play-session would predict reduction in anxiety symptoms at the 3-months follow-up. In line with these expectations, Table 5 shows that increases in exploration were significantly associated with lower anxiety scores 3 months later. For the avoidant/safety in-game play behaviours increases in ceiling light attempts and time spent hiding inside chests were significantly associated with higher anxiety scores 3 months later.

## Discussion

The video game *MindLight* translates evidence-based techniques into game mechanics that teach children how to cope with anxiety in a playful manner. The aim of the present study was to investigate whether children with elevated levels of anxiety improved in their anxiety levels through these game mechanics that were explicitly designed into *MindLight*. Based on the anxiety literature, two types of in-game play behaviours (i.e., “engaged” and “avoidant/safety”) that are most relevant to the intervention goals of *MindLight* were distinguished and coded during gameplay. First, contrary to what was expected, pretest anxiety scores were not associated with the different in-game play behaviours during the first play-session. Second, in line with our predictions, increases in one of the in-game engaged behaviours and decreases in the two avoidant/safety behaviours predicted lower anxiety scores at 3-months follow-up. Together, these findings suggest that mechanics related to exposure techniques predicted improvements in children’s anxiety symptoms.

### Associations between Pretest Anxiety Scores and In-Game Play Behaviours

Our finding that none of the in-game play behaviours, and the avoidant/safety behaviours in particular, were associated with pretest anxiety scores was unexpected. Because safety behaviours are an important maintenance process in anxiety disorders (Clark 1999), it was expected that children higher in anxiety would try to create more light by turning on ceiling lights and to hide more inside chests, because these objects provide a way to reduce and/or avoid exposure to fear events. The current study is part of an indicated prevention trial in which selection was based on scoring one standard deviation above the mean and clinical cases that already received mental health care were excluded. Therefore, the current finding might be due to a restricted range in anxiety scores. It might also be that the first play-session was standardized in such a way (with a lot of cut-scenes explaining the game) that little room was left for children to show very different in-game play behaviours. Nevertheless, it is promising that irrespective of their pretest anxiety scores, children started playing *MindLight* in a similar way because this strengthens the findings for the second hypothesis; changes in in-game play behaviours and the predicted improvements in anxiety symptoms are not due to associations between initial levels of anxiety and in-game play behaviours during the first session. Furthermore, the finding that none of the in-game play behaviours during the first session was associated with behaviours during the last session (except one association) is promising, because this suggests that *MindLight* is able to change the way in which children continued playing the game after the first session, enhancing opportunities to change anxiety symptoms.

Additional findings showed negative associations between several engaged and avoidant/safety in-game play behaviours during the first play-session, which provide support for the theory-based distinction between these two types of behaviours and at the same time supports the contention that the *MindLight Coding System-II* is able to distinguish these conceptually different in-game play behaviours.

### In-Game Play Behaviours Representing Exposure Predicted Improvements in Anxiety

Results showed that changes in in-game play behaviours representing therapeutic exposure techniques predicted improvements in anxiety symptoms 3 months later. Regarding the engaged in-game behaviours, exploring the fearful game-environment for longer periods of time predicted decreases in anxiety symptoms. The time spent on actual attempts to chase away these fearful stimuli did not predict changes in anxiety symptoms nor did the frequency of getting defeated by these fear events. Regarding the avoidant/safety in-game behaviours, more attempts to seek safety under a ceiling light and increases in time spent hiding inside chests (and therefore avoiding fearful stimuli) predicted higher anxiety symptoms 3 months later. These findings are in line with previous research on avoidant/safety behaviours during traditional therapy (Glenn et al. 2013; Hammond 2005; Morgan and Raffle 1999; Salkovskis et al. 1999; Price and Budzynski 2009). However, the present study also extends this research by investigating these predictors in a game-based intervention. The current findings suggest that avoidant/safety behaviors play a similar role in an applied video game as in traditional face-to-face therapy.

Moreover, relaxation during gameplay (indicated by no or total mindlight) did not predict changes in anxiety symptoms. It might be that relaxation during gameplay is not as important for improvements in anxiety as the exposure game mechanic. Recent evidence (published after *MindLight* was developed) suggests that the contribution of relaxation training to anxiety improvements in youth appears to be limited, although it might be that a higher dose of relaxation is needed to improve anxiety symptoms (Peris et al. 2015).

Most importantly, the present study provides a unique contribution to the field by demonstrating that changes in the interaction with the game mechanics in *MindLight* predicted real-world improvements in anxiety symptoms at the 3-months follow-up assessment (when children had not played the game for 3 months). These findings show that what children learned and practiced in the game led to actual changes in their real-life (self-reported) behaviours. Most CBT approaches are not able to close this gap (Granic et al. 2014; Kendall 2011) and only a few game-based interventions have examined these effects (e.g., Girard et al. 2013). Moreover, by including a long-term follow-up, the present study is one of the few longitudinal studies in the field of applied video games (Girard et al. 2013; Granic et al. 2014; Primack et al. 2012).

### Limitations and Suggestions for Future Research

There are a few limitations that should be mentioned when interpreting the findings and future research should attempt to overcome. The first limitation is that no in-game play behaviours representing the third technique (i.e., attention bias modification), that was initially incorporated in *MindLight*, were included in the current study. Because of how the game was designed, these behaviours were too dependent on the specific puzzle and the current location of the player and these locations varied widely across players, leaving comparisons across children unreliable. For example, a puzzle success on an easier puzzle would be different from a puzzle success on a more difficult puzzle, which requires more puzzle attempts and also the ability to distinguish different varieties of positive faces and negative faces. However, evidence for the effectiveness of attention bias modification in the literature is mixed. Some research has shown that attention bias modification is capable of reducing anxiety (Bar-Haim 2010; Bar-Haim et al. 2011; Hakamata et al. 2010), whereas other recent studies – that were published after *MindLight* was developed - failed to show this (for an overview see Clarke et al. 2014; Cristea et al. 2015). Clarke et al. (2014) argue that the studies finding no effects of attention bias modification failed to manipulate attention and therefore found no effects on the outcome measures. Future research should first test whether the puzzles in *MindLight* actually are able to modify attentional bias before in-game puzzle-solving behaviours are examined in relation to changes in anxiety.

A second limitation is that despite the fact that inactivity has good face validity for representing avoidant/safety behaviour because it reduces exposure to fear events and literally limits engagement with the game mechanics in *MindLight*, the inactivity code in the *MindLight Coding System-II* is not able to distinguish between actively avoiding the game or being inactive because the child is waiting for a research assistant to answer a question. Relatedly, the technical problem code might have been confounded too, because this code was used – but not exclusively – when the game was paused. It is possible that some children paused the game to actively avoid fear events in the game. Future research should try to separate these different behaviours, such that codes become as clear as possible and might show relations to changes in anxiety symptoms.

Finally, the current study did not test mediators or moderators. It is possible that associations between (multiple) in-game play behaviours and changes in anxiety symptoms are mediated or moderated by other factors, such as game expectancies, game experience, motivation, and enjoyment, which are known to be important predictors of treatment outcome (Castonguay et al. 1996; Ferguson and Olson 2013; Przybylski et al. 2010). Future research might want to examine these effects in a larger sample to get insight into why and for whom *MindLight* works best.

### Practical Implications

Notwithstanding the aforementioned limitations, the current study has three important implications. The first implication applies to clinical research on anxiety. Results showed that in-game behaviours representing the exposure principle predicted improvements in anxiety. These findings underscore the importance of this technique in anxiety disorders and show that applied games can be used to reduce anxiety symptoms. Using game-based interventions contribute to the movement of tailoring and personalizing treatments (Chorpita and Weisz 2009) by engaging and motivating children that might not like the rather didactic-based approach of traditional CBT-treatments (Gosch et al. 2006) and by providing rich practice opportunities for children that find it difficult to use newly-acquired knowledge in real-life situations (Granic et al. 2014).

The second implication relates to designs aimed at improving both gameplay experiences and intervention outcomes. Because results demonstrated that the *MindLight Coding System-II* is useful for distinguishing between engaged and avoidant/safety in-game play behaviours, the coding system could be integrated into the game itself. In-game play behaviours can then be tracked automatically and be used as a measure for determining children’s progress. Measurements of in-game behaviours can be used to dynamically adjust the game to the player’s actions, diverse needs and learning paces (Bakkes et al. 2012; Bakkes et al. 2014). For example, the game may provide more hiding spaces in the beginning of the game for more anxious children (cf. Milosevic and Radomsky 2008; cf. Rachman et al. 2008), or provide more exposures to fear events when children are able to stay relaxed and calm in the face of less/easier fear events. Additionally, such measurements could tell clinicians and researchers when and where children are encountering difficulties in the game, and provide opportunities to help at an early stage. Finally, measuring in-game behaviours provides feedback for the players, clinicians, researchers and future game-based interventions, without the biases and stigma that are associated with self-reports.

The third implication applies to future research and the development of game-based interventions. Using observational codes, the current study provided a first step in testing the effect of the game mechanics in *MindLight*; the exposure to fear events is an important game mechanic that predicted anxiety symptoms 3 months later. Because of the modularity of game design, games hold the immense potential to test mechanisms of change with tightly controlled experiments. The next step for future research could be to experimentally manipulate the game mechanics in *MindLight* to test their causal impact on improvements in anxiety. Different versions of the game could be played with and without the game mechanics. Such experimentally controlled studies contribute to the development of a toolbox of validated game mechanics that could be used in new games targeting anxiety symptoms or other psychopathologies with the same underlying deficits.

## Appendix 1

Description of the Video Game *MindLight*

*MindLight* incorporates three evidence-based techniques (i.e., relaxation, exposure, attention bias modification) to teach children how to cope with anxiety in a playful manner (see Table 1). To trigger real feelings of anxiety and to practice regulating these feelings, the game is set in grandma’s dark and decrepit mansion. Little Arty (the player) is left on the doorstep of grandma’s mansion that has been taken over by evil forces. It is his task to save grandma who succumbed to shadows. In the house Arty finds a magical headset (Teru) and together they can save grandma. However, the player needs to overcome his fears by using his own mind. Teru teaches how to use this “mindlight”, a beam of light at the end of an antenna attached to the magical hat which players need to play through the game.

Arty’s mindlight responds to the electroencephalogram (EEG) signals that the MindWave headset, a one-channel dry-sensor EEG headset the player is wearing, picks up. The headset detects and converts EEG signals (i.e., relative beta power and relative alpha power) into two continuous data streams representing relaxation and focused attention. These data streams are fed into the game (i.e., *neurofeedback training*) and control Arty’s mindlight and “mindbeam”. The strength of the mindlight and mindbeam are proportional to the strength of the relaxation and focused attention signals, respectively. Being more relaxed and focused makes it possible to effectively engage with the objects and evidence-based game mechanics in the game. When the player becomes more relaxed the mindlight will become stronger, providing more light inside the game environment which makes it easier to get through the game. When exposed to fear events, players need their mindlight to chase away or “decloak” these monsters. Some fear events that are decloaked will turn into a friendly kitten that will remind the player of past fears conquered. More focused attention leads to a stronger mindbeam, which is needed to unlock hiding spaces, turn on ceiling lights and to solve attention bias modification puzzles in which the player learns and is rewarded for focusing on positive faces rather than on negative faces.

The goal in *MindLight* is to save grandma by completing all puzzles, which will turn the lights back on in the house. To solve a puzzle, coins have to be found first to unlock the puzzle. Every puzzle requires a predetermined amount of coins (i.e., from 3 up to 7 coins) that corresponds to the number of faces in the puzzle. The amount of coins reflects the difficulty of the puzzle; a puzzle with more coins requires more effort than a puzzle with less coins because in the puzzle with more coins the positive face has to be distinguished from more negative faces.

## Footnotes

1 The effect sizes for the *MindLight* group were 0.60 from pre- to post-measurement, 0.75 at three-month follow-up, 1.07 at six-month follow-up, comparable to meta-analytic results on CBT (Mychailszyn et al. 2012) and Kendall’s Coping Cat in particular (Lenz 2015).

2 Significant disturbances included: [1] trainer was wearing the MindWave instead of the child, [2] child had to change laptops, which led to the situation that the child had to start over with the game, [3] technical problems or no connection with the MindWave for more than 50% of a specific session.

3 When a child was absent during the last or the fifth session, video data from the fifth or the fourth session was coded, respectively.

4 Although the MindWave headset provided a more continuous measure for relaxation through the EEG data, this data could not be matched to the video data. Therefore, the brightness of the mindlight as an indication for relaxation was coded manually.
